# Systematic Significance of Leaf Epidermal Features in *Holcoglossum* (Orchidaceae)

**DOI:** 10.1371/journal.pone.0101557

**Published:** 2014-07-01

**Authors:** Jie Fan, Runli He, Yinbo Zhang, Xiaohua Jin

**Affiliations:** 1 College of Chinese Medicine, Shanxi University of Traditional Chinese Medicine, Taiyuan, China; 2 College of Environmental Science and Resources, Shanxi University, Taiyuan, China; 3 State Key Laboratory of Systematics and Evolutionary Botany, Institute of Botany, Chinese Academy of Sciences, Beijing, China; University of Kent, United Kingdom

## Abstract

Determining the generic delimitations within Aeridinae has been a significant issue in the taxonomy of Orchidaceae, and *Holcoglossum* is a typical case. We investigated the phylogenetic utility of the morphological traits of leaf epidermis in the taxonomy of *Holcoglossum s.l*. by using light and scanning electron microscopy to analyze 38 samples representing 12 species of *Holcoglossum*, with five species from five closely related genera, such as *Ascocentrum*, *Luisia*, *Papilionanthe*, *Rhynchostylis* and *Vanda*. Our results indicated that *Holcoglossum* can be distinguished from the related genera based on cuticular wax characteristics, and the inclusion of *Holcoglossum himalaicum* in *Holcoglossum* is supported by the epidermis characteristics found by LM and SEM. The percentage of the tetracytic, brachyparacytic, and laterocytic stomata types as well as the stomata index and certain combinations of special wax types support infrageneric clades and phylogenetic relationships that have been inferred from molecular data. Laterocytic and polarcytic stomata are perhaps ecological adaptations to the strong winds and ample rains in the alpine region of the Hengduanshan Mountains.

## Introduction

Determining the generic delimitations within orchid subtribe Aeridinae is difficult and has been considered a “black pit” [1]–[7] and the genus *Holcoglossum* Schltr. is such an example [3], [5], [8]. The orchid genus *Holcoglossum* (Aeridinae, Vandeae, Orchidaceae) is comprised of approximately 17 species ranging from Malaysia, Thailand, Myanmar, Vietnam and northeastern Yunnan, and eight of which are endemic to China [9], and it is a key genus in the “*Vanda*-*Aerides*” alliance, which includes *Aerides*, *Ascocentrum*, *Ascolabium*, *Holcoglossum*, *Neofinetia*, *Papilionanthe*, *Penkimia*, *Seidenfadenia* and *Vanda* [10]. Since its establishment in 1919 [11], the genus has had several circumscriptions [3]–[5], [10]–[21]. Recent molecular phylogenetic studies have shed new light on the generic delimitation of *Holcoglossum* and its alliances. However, several different taxonomic treatments have been proposed based on molecular phylogenetic research [13], [20], [21]. A recent molecular phylogenetic study suggested that there are 17 species in *Holcoglossum s.l.*, including 14 species of *Holcoglossum s.s.*, and three species from its alliance, including *Ascocentrum himalaicum* (Deb. Sengupta & Malick) Christenson, *Ascolabium pumilum* (Hayata) Schltr., and *Penkimia nagalandensis* Phukan & Odyuo [13].

In addition to debates on the generic delimitation of *Holcoglossum*, several perspectives exist on the infrageneric taxonomy of this genus [10], [12], [13], [20], [21]. Christenson [10] divided *Holcoglossum* into two sections based on the flower number per inflorescence and plant form. Jin [12] proposed an infrageneric system based on floral characteristics, such as the callus, spur and stipe; this system has been supported by the results of recent molecular phylogenetic studies [13], [20]. However, four species in Jin's system, *H. omeiense* X. H. Jin & S. C. Chen, *H. lingulatum* (Averyanov) Averyanov, *H. wangii* Christenson and *H. kimballianum* (Rchb. f.) Garay in *H.* sect. *Holcoglossum*, share similar habits and floral characteristics, including a crested callus and long spur, are grouped into more distant clades in phylograms in molecular phylogenetic studies. The search for morphological synapomorphies for each monophyletic clade has been problematic because of widespread homoplasy [13], [20]. And anatomical studies of leafless Vandeae have revealed an extreme homoplasy in leaf and root characteristics with no clear evolutionary trend, even among genera [23].

In an attempt to overcome these difficulties, previously underexplored morphological characteristics are now being studied in more depth, such as characteristics of pollinia and leaf epidermis. Although pollinia characteristics (number and aperture) are largely homoplasious for Aeridinae in total, they are often useful at the generic and/or infrageneric level. For instance, *Neofinetia* and *Holcoglossum* have porate pollinia, whereas *Vanda, Rhyncostylis, Aerides* and *Trudelia* have cleft pollinia [7], [13], [22]. The mature leaf epidermal characteristics, such as the pattern of epidermal cells, type of stomata, shape of guard cell pairs and ornamentation of cuticles, are considered useful in the phylogenetic studies at the level of genera or subgenera in angiosperms [24], [25]. However, the use of such characteristics varies in importance in the phylogenetic study of Orchidaceae, and it has played a minor role in studies of leaf anatomy in Orchidaceae in recent years [26]–[31]. Some authors suggest that the development of the stomata is required to understand the phylogenetic importance of stomatal characteristics because different development pathways may result in the same stomatal type [27]. However, the stomata type as a systematic characteristic has been used to divide the Orchidaceae taxonomy and Orchidaceae into four divisions: Anomocyticeae, Acyticeae, Cyclocyticeae and Paracyticeae [32]. Tomlinson [27] suggested that the stomata complex has value as a systematic characteristic only in combination with a variety of other characterristics and only in groups that are already known to be natural.

Recent studies have indicated that the ontogeny of stomata in *Holcoglossum* and its relatives is the perigenous, which is similar to most of Orchidaceae, and tetracytic type of stomata (four somewhat equally sized subsidiary cells around the stoma) is the most popular stomatotype [27], [29], [30]. Furthermore, *Holcoglossum* is a natural group suggested by molecular phylogenetic studies [13], [20]. Hence, it is reasonable to consider the phylogenetic significance of the mature leaf characteristics without studying the development of stomata in *Holcoglossum*. The aim of the present study is to investigate whether the characteristics of mature leaf epidermis can provide additional taxonomic and phylogenetic information for the genus *Holcoglossum* and the infrageneric clades that have been suggested by recent results of molecular phylogenetics.

## Results

The leaf epidermal characteristics are listed in Tables 1, 2, 3. The molecular phylogeny of the *Holcoglossum* infrageneric clades mentioned below includes three clades from Xiang et al. [13]: TC (tropic clade, including *H. amesianum* (Rchb. f.) Christenson, *H. subulifolium* (Rchb. f.) Christenson, *H. wangii*, *H. kimballianum, H. nagalandense* (Phukan & Odyuo) X.H. Jin, and *H. himalaicum* (Deb, Sengupta & Malick) Aver.), AC (the temperate alpine clade, including *H. flavescens* (Schltr.) Z.H. Tsi, *H. rupestre* (Hand.-Mazz.) Garay, *H. sinicum* Christenson, *H. weixiense* X.H. Jin & S.C. Chen, *H. tsii* T. Yukawa and *H. nujiangense* X.H. Jin & S.C. Chen), and HC (clade located between TC and AC, including *H. omeiense*, *H. lingulatum, H. quasipinifolium* (Hayata) Schltr., and *H. pumilum* (Hayata) X.H. Jin).

**Table 1:** Leaf epidermal characteristics of *Holcoglossum*, its relatives and outgroup under LM.

Taxa	Shape of cells	Pattern of anticlinal walls	Stomatal apparatus	Size of stoma (L/W)	Stoma index (%)	Stoma grouping (%)	Figures
	(Ad/Ab)						
*Holcoglossum nujiangense*	Pol(4)/Pol(4)	Str/Str	Exist/Exist	1.2/0.9	2.6/2.8	0/28.6	Fig 3, B–C
*H. weixiense*	Pol(4)/Pol(4)	Str/Str	Exist/Exist	1.4/1.2	2.1/2.8	30.8/22.9	Fig 3, D–E
*H. sinicum*	Pol(4)/Pol(4)	Str/Str	Exist/Exist	1.1/1.0	2.2/3.1	7.7/11.3	Fig 2, G–H
*H. rupestre*	Pol(4)/Pol(4)	Str/Str	Exist/Exist	1.1/1.0	2.0/2.6	31.3/23.6	Fig 2, I; Fig 3 A
*H. flavescens*	Pol(4–6)/Pol(4–6)	Str/Str	Exist/Exist	0.9/1.0	2.0/2.6	7.1/21.4	Fig 2, E–F
*H. omeiense*	Pol(4–6)/Pol(4–6)	Str/Str	Exist/Exist	-/0.9	-/4.3	-/19.8	Fig 3, H–I
*H. lingulatum*	Pol(4–6)/Pol(4–6)	Str/Str	Exist/Exist	-/1.1	-/4.0	-/16.5	Fig 3, F–G
*H. himalaicum*	Pol(6)/Pol(6)	Str/Str	Exist/Exist	1.3/1.1	2.0/2.1	0/0.6	Fig 4, C–D
*H. wangii*	Pol(6)/Pol(6)	Str/Str	Exist/Exist	0.9/1.0	0.6/0.7	0/0	Fig 1, G–H
*H. kimballianum*	Pol(6)/Pol(6)	Str/Str	Exist/Exist	1.2/1.3	0.6/0.7	0/0	Fig 1, E–F
*H. amesianum*	Pol(6)/Pol(6)	Str/Str	Exist/Exist	1.0/1.0	1.9/2.2	0.5/0.5	Fig 1, A–B
*H. subulifolium*	Pol(6)/Pol(6)	Str/Str	Exist/Exist	1.1/1.1	2.1/1.4	0/0	Fig 1, C–D
*Papilionanthe biswasiana*	Pol(4–6)/Pol(4–6)	Str-arc/Str-arc	Exist/Exist	1.3/1.3	7.5/7.5	25.0/25.0	Fig 1, I
*Ascocentrum ampullaceum*	Pol(4–6)/Pol(6)	Str-arc/Str-arc	Exist/Exist	-/1.4	-/2.5	-/3.1	Fig 2, A–B
*Vanda pumila*	Pol(4–6)/Pol(4)	Str/Str	Absent/Exist	0/1.3	0/2.6	0/0	Fig 2, C–D
*Rhynchostylis retusa*	Pol(6)/Pol(6)	Str/Str	Exist/Exist	1.3/1.4	3.8/4	0/1.5	Fig 4, A–B
*Luisia magniflora*	Pol(4–6)/Pol(4–6)	Str-arc/Str-arc	Exist/Exist	1.2/1.2	3.1/3.1	5.8/5.8	Fig 4, E

Pol(4), polygon (quadrilateral); Pol(6), polygon (hexagonal); Pol(4–6), polygon (quadrilateral to hexagonal); Str, straight; Str-arc, straight to arched; Ad/Ab, adaxial epidermis/abaxial epidermis; -, no data analysis.

**Table 2:** Stomatotypes and substomatotypes of *Holcoglossum*, its relatives and outgroup under LM.

Taxa	Stomatotype (%) Ad/Ab														
	Tetra	Para						Latero				Polar			
		Hemipara	Brachypara	1+2	2+2	3+(1–4)	Total	1+2	2+2	3+(1–4)	Total	5(1+2)	6(2+2)	≥6(3+{?1–4}?)	Total
*Holcoglossum nujiangense*	9.1/0	0/0	0/0	9.1/0	0/0	0/0	9.1/0	18.2/42.9	27.3/28.6	36.4/0	81.8/71.4	0/14.3	0/0	0/14.3	0/28.6
*H. weixiense*	8.7/0	0/0	4.3/0	17.4/16.1	17.3/3.2	0/0	39.1/19.4	13.0/25.8	30.4/12.9	8.7/16.1	52.1/54.8	0/6.5	0/6.5	0/12.9	0/25.8
*H. sinicum*	9.1/9.1	0/0	18.2/3.0	18.2/3.0	4.5/0	0/3.0	40.9/9.1	18.2/12.2	0/18.2	9.1/15.2	27.3/45.6	0/9.1	9.1/6.1	13.6/21.2	22.7/36.4
*H. rupestre*	4.2/4.2	0/0	20.8/3.8	12.5/7.7	4.2/0	0/0	37.5/11.5	8.0/25.0	8.3/5.8	20.8/26.9	37.1/57.7	4.2/7.7	4.2/0	12.5/19.2	20.8/26.9
*H. flavescens*	14.3/5.0	0/0	8.6/3.0	14.3/7.0	2.9/4.0	5.7/3.0	31.5/17.0	8.6/14.0	5.7/9.0	0/7.0	14.3/30.0	22.9/28.0	5.7/10.0	11.4/10.0	40.0/48.0
*H. omeiense*	-/8.4	0/0	-/3.4	-/9.2	-/3.4	-/0	-/15.7	-/13.8	-/9.2	-/9.2	-/32.2	-/19.5	-/10.3	-/13.8	-/43.7
*H. lingulatum*	-/11.5	0/0	-/9.8	-/1.6	-/1.7	-/0	-/13.1	-/18.1	-/5.0	-/4.9	-/27.9	-/29.5	-/4.9	-/13.1	-/47.5
*H. himalaicum*	50.7/68.6	0/0	30.7/21.4	5.3/0	0/0	0/0	36.0/21.4	6.7/5.7	2.7/0	2.7/1.4	12.1/7.1	1.3/0	0/2.9	0/0	1.3/2.9
*H. wangii*	46.2/37.1	0/0	15.3/20.0	0/5.7	0/0	0/0	15.4/25.7	15.3/20.0	7.7/8.6	7.7/0	30.8/28.6	7.7/8.6	0/0	0/0	7.7/8.6
*H. kimballianum*	58.3/31.2	0/0	37.5/30.3	0/0.9	0/1.8	0/0	37.5/33.0	0/8.3	0/4.6	0/0	0/12.9	4.2/14.7	0/4.6	0/3.7	4.2/22.9
*H. amesianum*	43.1/31.1	0/0	45.1/33.3	0/0	0/0	0/0	45.1/33.3	7.8/22.2	2.0/2.2	0/2.2	9.8/26.7	2.0/6.7	0/2.2	0/0	2.0/8.9
*H. subulifolium*	63.8/56.5	0/0	23.4/27.2	0/0	0/0	0/0	23.4/27.2	0/14.1	0/1.1	0/0	0/15.2	12.8/1.1	0/0	0/0	12.8/1.1
*Papilionanthe biswasiana*	0/0	0/0	38.4/38.4	23.1/23.1	23.1/23.1	0/0	38.4/38.4	15.4/15.4	0/0	0/0	61.5/61.5	0/0	0/0	0/0	0/0
*Ascocentrum ampullaceum*	-/14.9	0/0	-/7.5	-/10.4	-/4.5	-/0	-/22.4	-/31.3	-/13.4	-/1.5	-/46.2	-/9.0	-/6.0	-/1.5	-/16.5
*Vanda pumila*	0/4.5	0/0	0/22.7	0/0	0/4.5	0/0	0/27.2	0/18.2	0/22.7	0/4.5	0/45.4	0/0	0/13.6	0/9.1	0/22.7
*Rhynchostylis retusa*	30.0/26.5	0/0	60.0/63.8	0/0	0/0	0/0	60.0/63.8	10.0/9.6	0/0	0/0	10.0/9.6	0/0	0/0	0/0	0/0
*Luisia magniflora*	0/0	6.3/6.3	84.8/84.8	0.9/0.9	0.4/0.4	0/0	92.4/92.4	4.9/4.9	1.3/1.3	0/0	6.2/6.2	1.3/1.3	0/0	0/0	1.3/1.3

**Figure 1:**
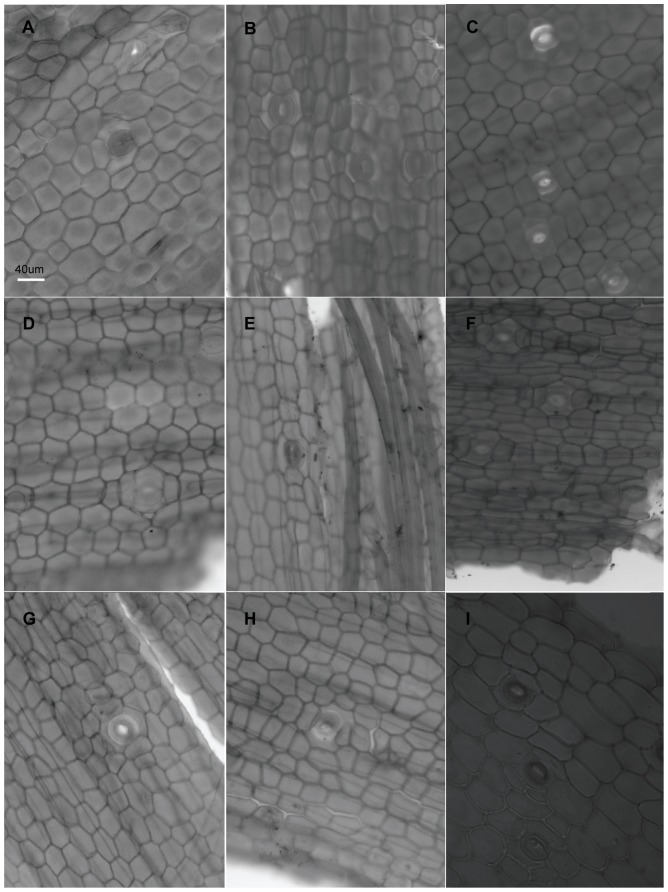
Characteristics of the epidermal cells (LM). A–B. *Holcoglossum amesianum*. A, adaxial, tetracytic; B, abaxial, brachyparacytic and laterocytic 1+2. C–D. *H. subulifolium*. C, adaxial, tetracytic and brachyparacytic; D, abaxial, tetracytic. E–F. *H. kimballianum*. E, adaxial, tetracytic; F, abaxial, tetracytic. G–H. *H. wangii*. G, adaxial, tetracytic; H, abaxial, tetracytic. I. *Papilionanthe biswasiana*, brachyparacytic and laterocytic 1+2. B–I have the same scale bars as in A.

Ad/Ab, adaxial epidermis/abaxial epidermis; tetra, para, latero and polar, represent tetracytic, paracytic, laterocytic and polarcytic respectively; para 1+2 indicates the distribution of the subsidiary cells (in this case, 3 subsidiary cells with1 on one lateral side of the stoma and 2 on the other side); latero 1+2 (same meaning as para 1+2); polar 5(1+2) indicates the distribution of the subsidiary cells (in this case, 5 subsidiary cells with 2 polar not inlcluded in the name of type and 3 lateral,1+2 represents 3 lateral with same meaning as para 1+2); -, no data analysis. Each species is represented in Figure 1.

**Table 3:** Wax types of the leaf epidermides of *Holcoglossum*, its relatives and outgroup under SEM.

Taxa	Wax Types(Ad)									Wax Types(Ab)								Figures
	1	2	3	3*	3**	4	4*	5	6	1	2	3	3*	3**	4	4*	5	
*Holcoglossum sinicum*	-	▴	-	-	▴	-	-			-	▴	-	-	▴	-	-	-	Fig 7, E–F
*H. nujiangense*	-	▴	-	▴	-	-	-	▴		-	▴	-	▴	-	▴	-	▴	Fig 7, C–D
*H. flavescens*	-	▴	-	▴	-	▴	-	▴		-	▴	-	-	▴	▴	-	▴	Fig 7, A–B
*H. omeiense*	-	▴	-	-	▴	▴	-	-		-	▴	-	-	▴	▴	-	-	Fig 6, E–F
*H. lingulatum*	-	▴	-	-	▴	▴	-	-		-	▴	-	-	▴	▴	-	-	Fig 6, C–D
*H. himalaicum*		▴							▴		▴	▴			▴			Fig 9, C–D
*H. kimballianum*	-	▴	-	▴	-	▴	-	-		-	▴	-	▴	-	▴	-	-	Fig 6, A–B
*H. wangii*	-	▴	▴	-	-	▴	-	-		-	▴	▴	-	-	▴	-	-	Fig 5, E–F
*H. subulifolium*	-	▴	▴	-	-	▴	-	-		-	▴	-	▴	-	▴	-	-	Fig 5, C–D
*H. amesianum*	-	▴	-	▴	-	▴	-	-		-	▴	-	▴	-	▴	-	-	Fig 5, A–B
*Papilionanthe biswasiana*	-	▴	-	-	▴	-	▴	-		-	▴	-	-	▴	-	▴	-	Fig 8, E
*Ascocentrum ampullaceum*	▴	▴	-	-	-	-	-	-		▴	▴	-	-	-	-	-	-	Fig 8, A–B
*Vanda pumila*	▴	▴	-	▴	-	-	-	-		▴	▴	-	-	-	-	-	-	Fig 8, C–D
*Rhynchostylis retusa*	▴	▴				-/▴				▴	▴							Fig 9, A–B
*Luisia maniflora*	▴	▴								▴	▴							Fig 9, E

Ad/Ab, adaxial epidermis/abaxial epidermis; 1, crusts; 2, granules; 3, sparse platelets; 3*, medium platelets; 3**, dense platelets; 4, flat rodlets; 4*, distinct rodlets; 5, threads; 6, membrancous platelets; ▴, with this character; -, without this character.

### Epidermal cells

**Figure 2:**
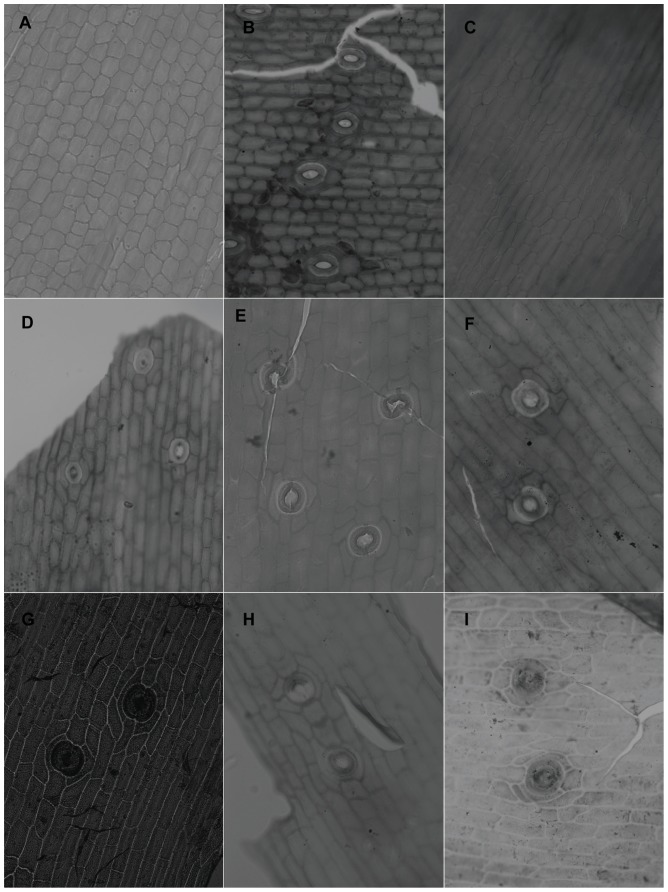
Characteristics of the epidermal cells (LM). A–B. *Ascocentrum ampullaceum*. A, adaxial; B, abaxial, polarcytic 6 (2+2) and polarcytic 5 (1+2). C–D. *Vanda pumila*. C, adaxial; D, abaxial, brachyparacytic and polarcytic 6 (2+2). E–F. *Holcoglossum flavescens*. E, adaxial, tetracytic, polarcytic 5(1+2) and polarcytic 6 (2+2); F,abaxial, polarcytic 5 (1+2) and laterocytic 3+4. G–H. *H. sinicum*. G, adaxial, paracytic and laterocytic 1+2; H, abaxial, polarcytic 5 (1+2) and polarcytic 5 (1+2). I. *H. rupestre*, adaxial, polarcytic 5 (1+2). Scales are the same as in Fig 2.

**Figure 3:**
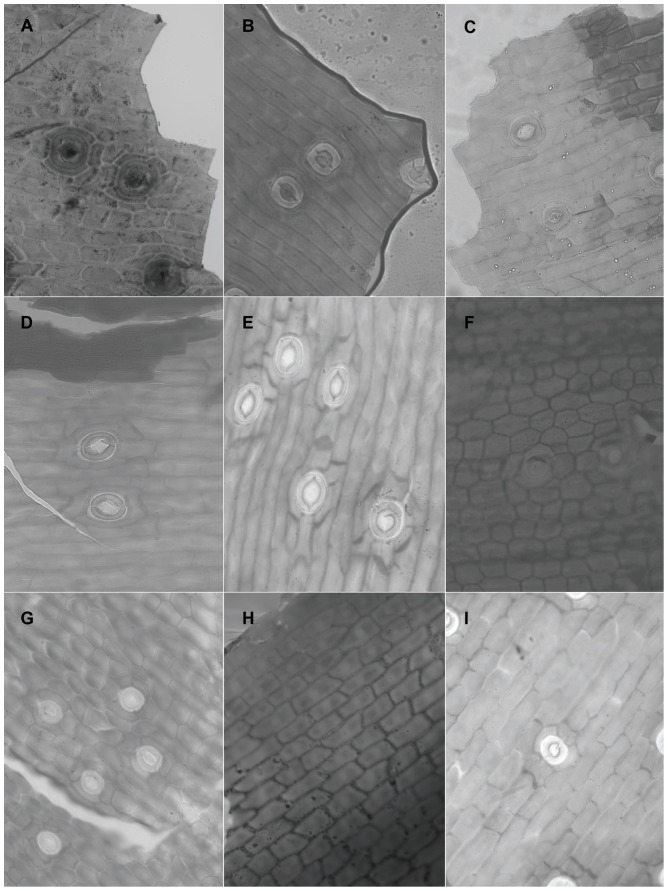
Characteristics of the epidermal cells (LM). A. *Holcoglossum rupestre*, abaxial, tetracytic and polarcytic 6(1+3). B–C. *H. nujiangense*. B, adaxial, laterocytic 1+2; C, abaxial, polarcytic 6 (2+2). D–E. *H. weixiense*. D, adaxial, tetracytic and laterocytic 2+3; E, abaxial, paracytic 2+2, laterocytic 1+3, polarcytic 6(1+3) and polarcytic 8(3+3). F–G. *H. lingulatum*. F, adaxial, paracytic 2+2; G, abaxial, tetracytic, brachyparacytic, polarcytic 5 (1+2) and polarcytic 6(2+2). H–I. *H. omeiense*. H, adaxial; I, abaxial, tetracytic. Scales are the same as in Fig 2.

**Figure 4:**
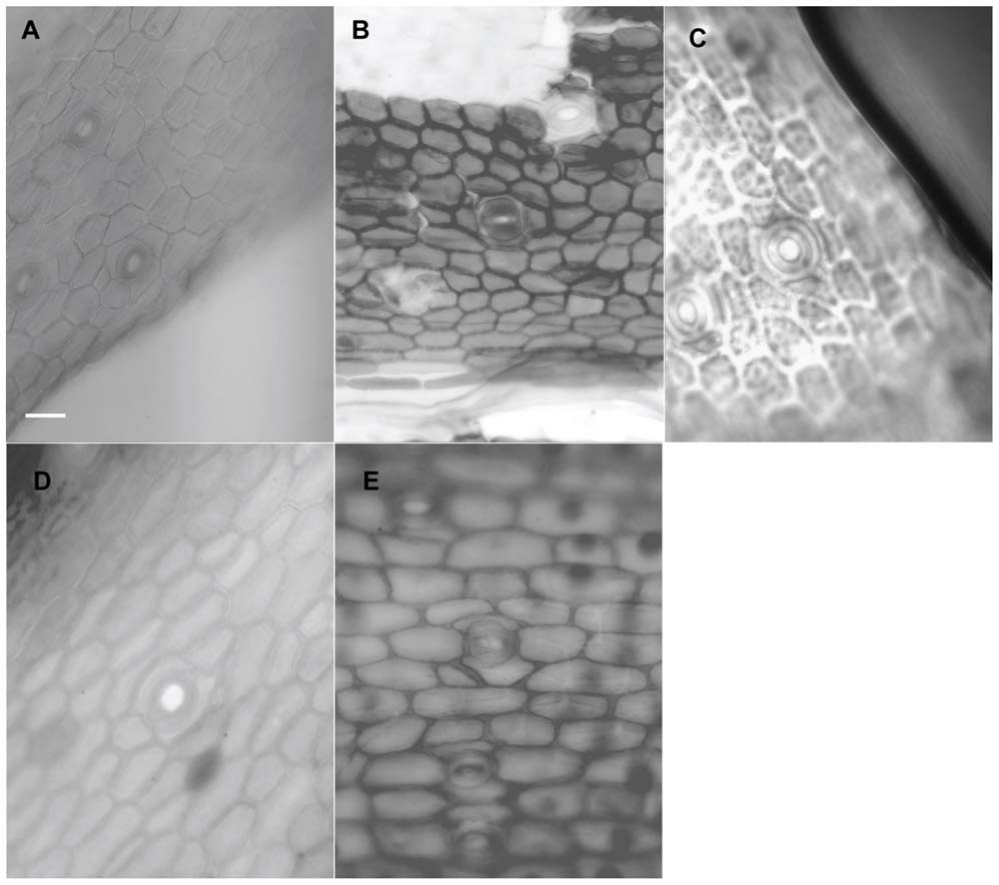
Characteristics of the epidermal cells (LM). A–B. *Holcoglossum himalaicum*. A, adaxial, tetracytic; B, abaxial, tetracytic. C–D. *Rhynchostylis retusa*. C, adaxial, tetracytic; D, abaxial, tetracytic. E. *Luisia magniflora*. brachyparacytic. B–E have the same scale bars as in A.

**Figure 5:**
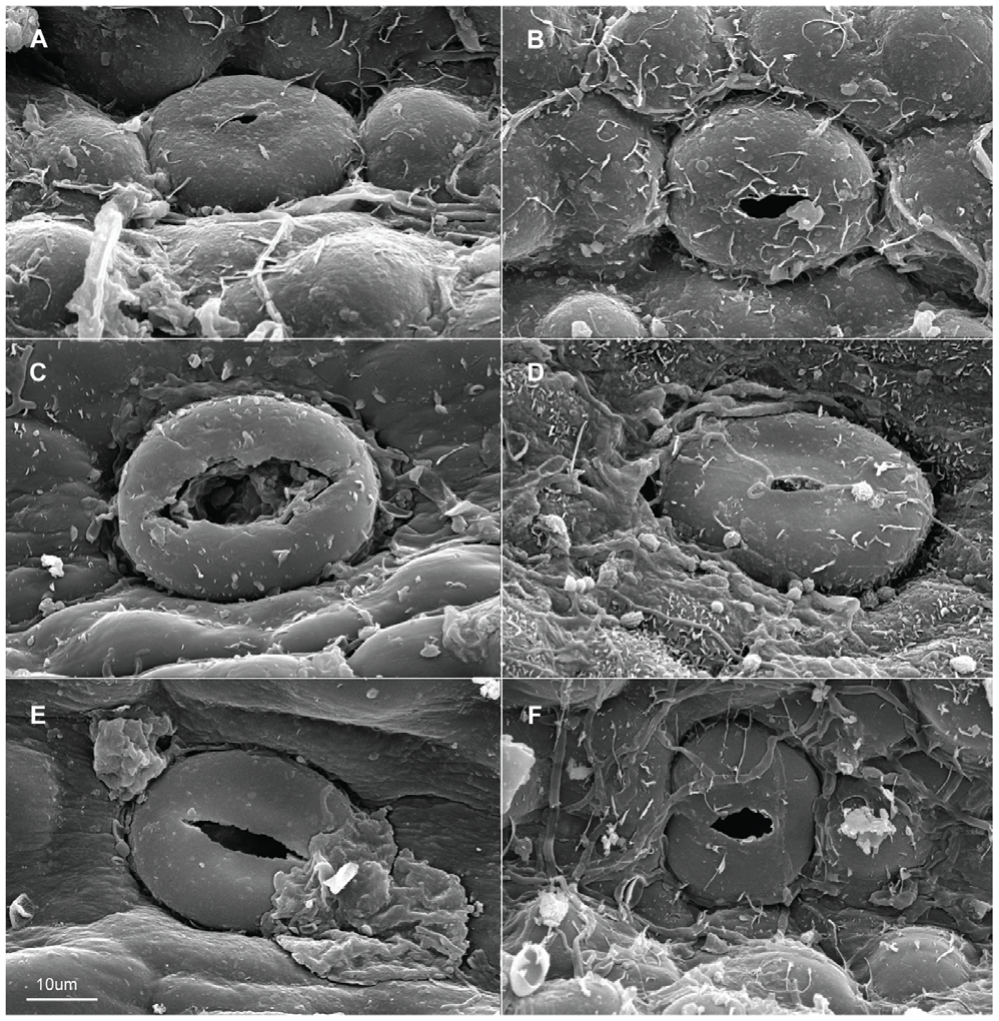
Characteristics of the epidermal cells (SEM). Adaxial on the left, abaxial on the right. A–B. *Holcoglossum amesianum*; C–D. *H. subulifolium*; E–F. *H. wangii*. A–D, F have the same scale bars as in E.

**Figure 6:**
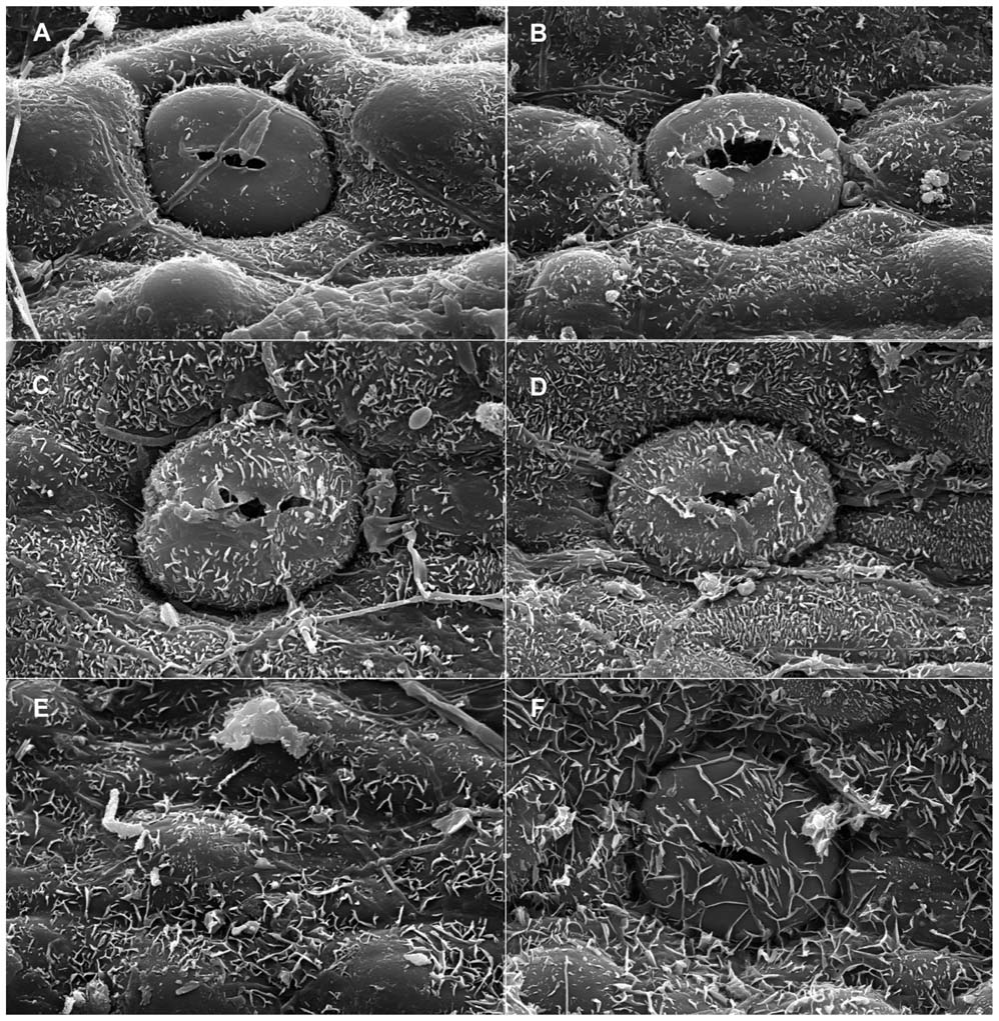
Characteristics of the epidermal cells (SEM). Adaxial on the left, abaxial on the right. A–B. *Holcoglossum kimballianum*; C–D. *H. lingulatum*; E–F. *H. omeiense*. Scales are the same as in Fig 6.

**Figure 7:**
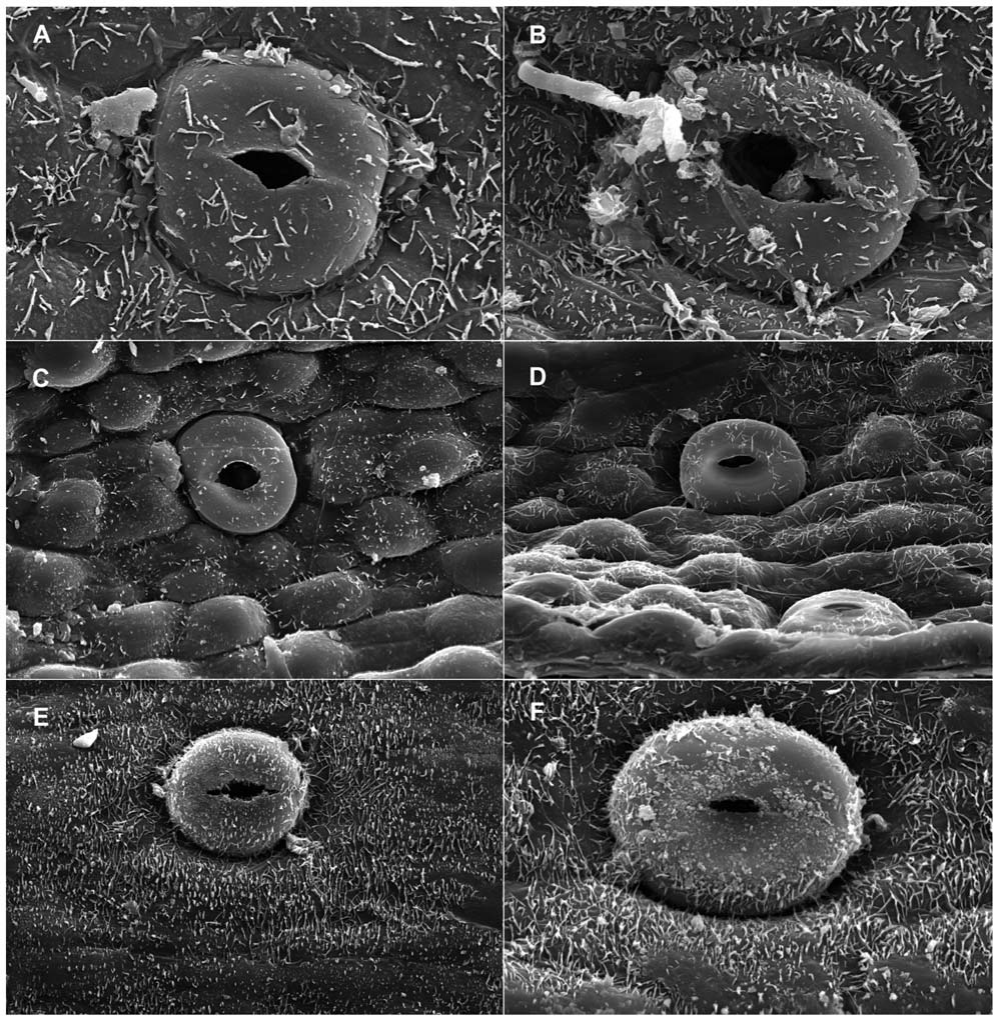
Characteristics of the epidermal cells (SEM). Adaxial on the left, abaxial on the right. A–B. *Holcoglossum flavescens*; C–D. *H. nujiangense*; E–F. *H. sinicum*. Scales are the same as in Fig 6.

**Figure 8:**
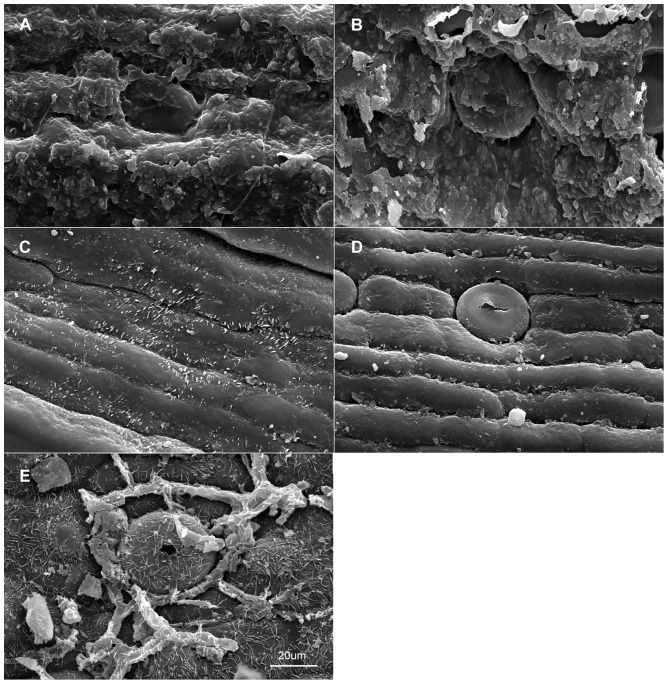
Characteristics of the epidermal cells (SEM). Adaxial on the left, abaxial on the right. A–B. *Ascocentrum ampullaceum*; C–D. *Vanda pumila*; E. *Papilionanthe biswasiana*. A–D have the same scale bars as in E.

**Figure 9:**
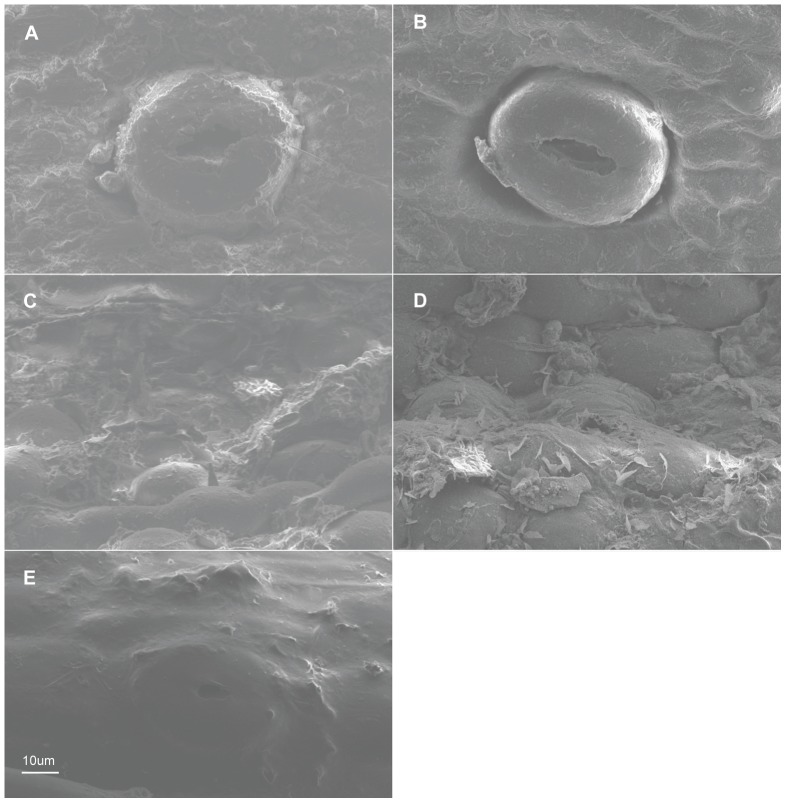
Characteristics of the epidermal cells (SEM). Adaxial on the left, abaxial on the right. A–B. *Rhynchostylis retusa*; C–D. *Holcoglossum himalaicum*; E. *Luisia magniflora*. A–D have the same scale bars as in E.

When viewed under LM, the epidermal cells on a single leaf including cells connected to stomata and those around it, have the same shape although narrower in some depressed strip areas that are parallel to the length of the leaf, and it is usually polygonal in form in all of the sample materials in this study. In *Holcoglossum*, the shape is hexagonal in the TC clade, quadrilateral to hexagonal in the HC clade and *H. flavescens* of the AC clade, and quadrilateral in all of the other species of the AC clade (Figs. 1, 2, 3, 4). When examined under SEM, the anticlinal walls of the epidermal cells are obscure or invisible under thick waxes (Figs. 5, 6, 7, 8, 9).

There are six wax types and six varieties: 1, crusts; 2, granules; 3, sparse platelets; 3*, medium platelets; 3** dense platelets; 4, flat rodlets; 4* distinct rodlets; 5, threads; and 6, membranous platelets. Normally, the wax characteristics of the abaxial (Ab) surface are more distinct than those of the adaxial (Ad) surface. Flat rodlets on the abaxial surface of the leaves and threads are only found in *Holcoglossum*; whereas granules are present in all of the samples (Table 3). In the genus *Holcoglossum*, flat rodlets are mainly found in the TC and HC clades on both surfaces of the leaves except for *H. himalaicum* on the abaxial surface (Figs. 5, 6; Fig. 7. A, B, D; Fig 9. D); threads are present in two species of the AC clade on both surfaces of the leaves (Fig. 7. A–D); dense platelets are predominant in the HC clade and some species of the AC clade on both surfaces of the leaves except for *H. flavescens* on the abaxial surface (Fig. 6. C–F and Fig. 7. B, E, F); and sparse or medium platelets present mainly in the TC clade on both surfaces of the leaves except for *H. himalaicum* on the abaxial surface (Fig. 5. A–F; Fig. 6. A, B; Fig. 9. D). Trichomes are found in *H. himalaicum* and *Luisia magniflora* Z. H. Tsi & S. C. Chen (Fig. 9. C) and secretory cells are occasionally seen in a few species, such as in the adaxial leaf surface of *H. subulifolium*.

### Stomatal apparatus

Stomata are present on both surfaces in all of the samples of *Holcoglossum, Rhynchostylis retusa* (L.) Bl. and *Ascocentrum ampullaceum* (Roxb.) Schltr., although rare stomata are present on the Ad surface of *H. omeiense*, *H. lingulatum* and *A. ampullaceum* (Figs. 5, 6, 7; Fig. 8. A, B; Fig. 9. A, B). They are only present on the Ab surface in *Vanda pumila* Hook. f. (Fig. 8. D). In *Papilionanthe biswasiana* (Ghose et Mukerjee) Garay and *Luisia magniflora*, stomata are presented on the entire leaf surface because of its terete leaf (Fig. 8. E and Fig. 9. E).

Stomatal clustering (mostly two stomata) is mainly found in the AC and HC clades and scarcely found in the TC clade of genus *Holcoglossum* (Fig. 3. A, B, E). Stomatal clustering consists of “non-contiguous clusters” formed by groups of stomata that do not have contact with each other (they are separated by the subsidiary cells) [33].

The guard cells are on the same level with the epidermis in the TC clade, are above the epidermis in the AC clade and at a medium level in the HC clade (Figs. 5, 6, 7 and Fig. 9. C, D).

### Stomata types

The stomata on a single leaf conform to several types, including tetracytic, paracytic, laterocytic or polarcytic. Other very rare stomatotypes found in several species are not included in the total of the stomatotypes; these stomatotypes include the hexacytic on the Ab surface of *A. ampullaceum*, *H. lingulatum*, and *H. flavescens* and Ad surface of *H. weixiense* and the stephanocytic type on both surfaces of *H. flavescens. H. rupestre*, and *P. biswasiana* and Ab surface of *H. lingulatum*. The determination of the extent of the variability, the predominant stomatotype and the proportion of these types is required so that the stomatal characteristics can be properly correlated with other characteristics, which would be useful for classification purposes [34].

In *Holcoglossum*, the tetracytic type is present in all of the samples except on the Ab surface of *H. nujiangense* and *H. weixiense*. This type has value scopes on the Ab surface of 31.1–68.6% in TC, 0–9.1% in AC and 8.4–11.5% in HC. The brachyparacytic type on the Ab surface has similar value scopes, with 20.0–38.4% in TC, 0–3.8% in AC and 3.4–9.8% in HC. The laterocytic type on the Ab surface has a reverse tendency with 12.9–28.6% in TC, 27.9–32.2% in HC, and 30.0–71.4% in AC (Table 2). Because there is infrequent stomata on the Ad surface of *H. lingulatum* and *H. omeiense*, the percentage of the stomatal types in these two species has little significance. Except for these two species, the percentage of tetracytic type on the Ad surface has value scopes of 43.1–63.8% in TC and 4.2–12.2% in AC (Table 2).

### Stomatal index

The scope of the stomatal index is 0–7.5%/0.7%–7.5% (Ad/Ab). The stomatal index on the Ab surface is divided into two groups, and *H. wangii*, *H. kimballianum*, *H. himalaicum, H. omeiense* and *H. lingulatum* are divided into two clades with the former three species in the TC clade (0.7%–2.2%) and the latter two in the HC clade (4.0%–4.3%).

## Discussion

### Systematic significance of the leaf epidermis in the generic delimitation and the subdivision of the genus *Holcoglossum*

In this study, we found that the wax type is a useful taxonomic characteristic that can distinguish *Holcoglossum* from its relatives and define infrageneric clades that are recognized in molecular studies [13], [20]. Flat rodlets, threads or only dense platelets in addition to granules on the Ab surface are characteristics of *Holcoglossum*. Our results lend support to the broad taxonomic treatment of *Holcoglossum* with infrageneric systems. Our results indicate that stomatal and other epidermal features in the Ab surface of the leaves are constant within species and represent good characteristics for taxonomic and phylogenetic studies.

### Infrageneric evolutionary directions and ecological adaptions to the environment

**Figure 10:**
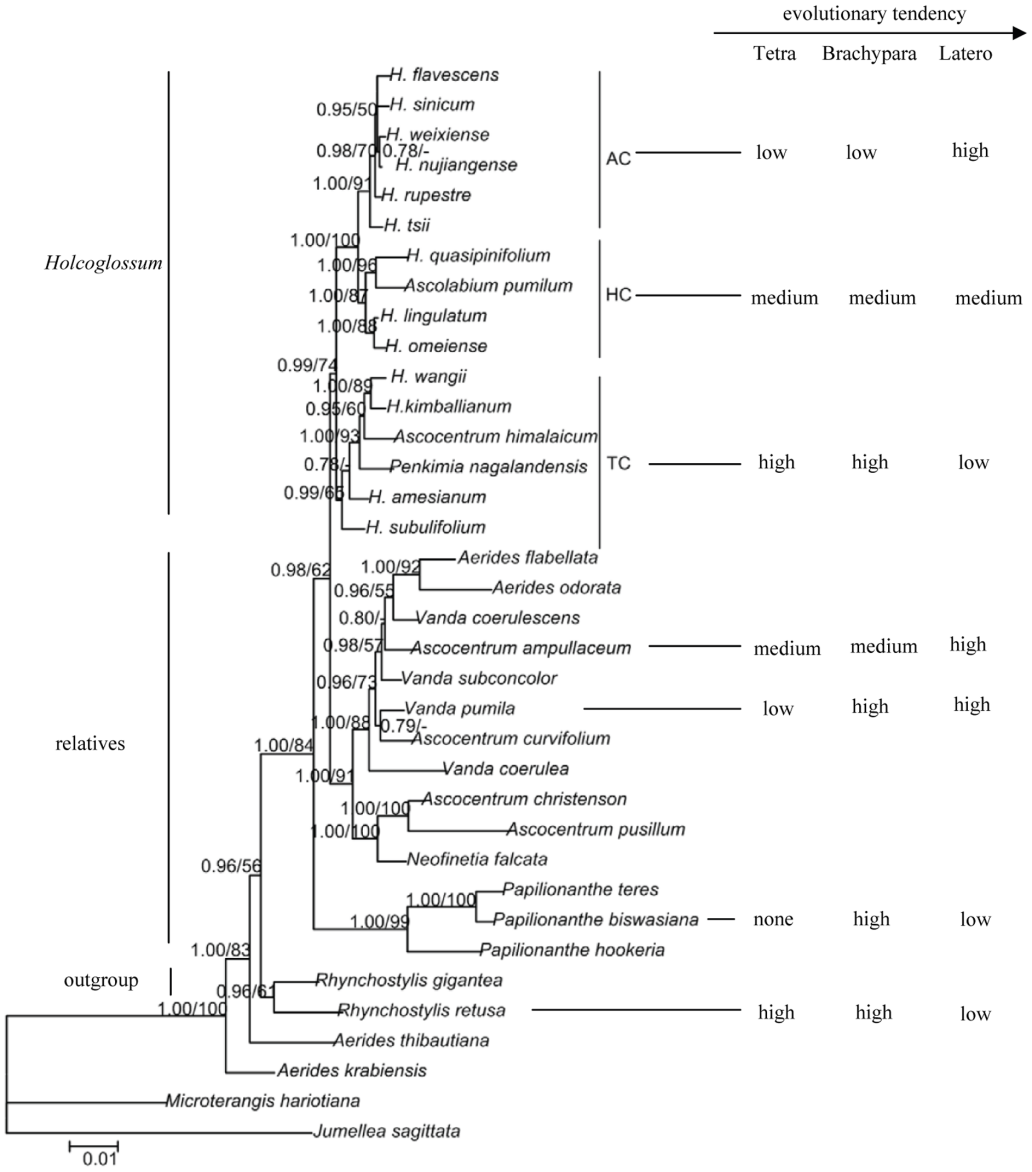
Molecular phylogeny presented by Xiang et al. (2012) with stomatotypes of the Ab surface arranged accordingly. Tetra, brachypara, latero represent tetracytic, brachyparacytic, and laterocytic, respectively.

The percent of the stomatotypes on the Ab surface of *Holcoglossum* has an obvious infrageneric evolutionary tendency, with the percentage of the tetracytic and brachyparacytic types decreasing in the direction of TC>HC>AC, although there are small intersections between HC and AC clades, whereas the percentage of the laterocytic type is in the reverse order with the highest value in the AC clade (Fig. 10). Using *Rhynchostylis retusa* as outgroup, our results indicated that a high percentage of tetracytic and brachyparacytic types and low percentage of laterocytic types are more basal (Fig. 10). The tetracytic type is a relatively simple type with one subsidiary cell on each lateral side of the stoma, whereas the laterocytic type is a comparatively complex type with more than 2 subsidiary cells on one lateral side or both lateral sides of the stoma. Our results are consistent with Williams' opinion [29] that more than two subsidiary cells are more evolutionarily derived than only two subsidiary cells, with more than four subsidiary cells only found in the most advanced orchids, such as species of *Trichocentrum* and *Erycina*. Species of *Trichocentrum* have fleshy or terete leaves, similar to the channeled semi-terete leaves of *Holcoglossum* [29].

It has been reported that the lateral wall of the meristemoid is never intersected by a transverse wall of the two adjacent files in the development of a stoma complex and the resultant stoma complex rarely occurs in the majority of the monocotyledons except *Luisia* [27]. It is interesting that *Holcoglossum* is also an exception with the anticlinal walls perpendicular to the long axis of the pore, which is found in its laterocytic and polarcytic types. The lateral wall of the meristemoid never intersected by a transverse wall of the two adjacent files has the value minimizing mechanical stresses in a meristem which enlarge chiefly in longitudinal direction [27]. We infer that the laterocytic and polarcytic types in *Luisia* and *Holcoglossum* are perhaps an adaptation to the longitudinal and intersected directions' growth of the tereted-leaves or semitereted-leaves. However, the percentage of laterocytic or polarcytic types is different between these two genera in this study, and it is much higher in *Holcoglossum* than in *Luisia*. The highest value is found in the AC clade of *Holcoglossum*, which is restricted to the Hengduan Mountains in southwestern China, and our results suggested that laterocytic or polarcytic is perhaps related to the cool winds and the heavy rains found in the high mountains [32].

**Figure 11:**
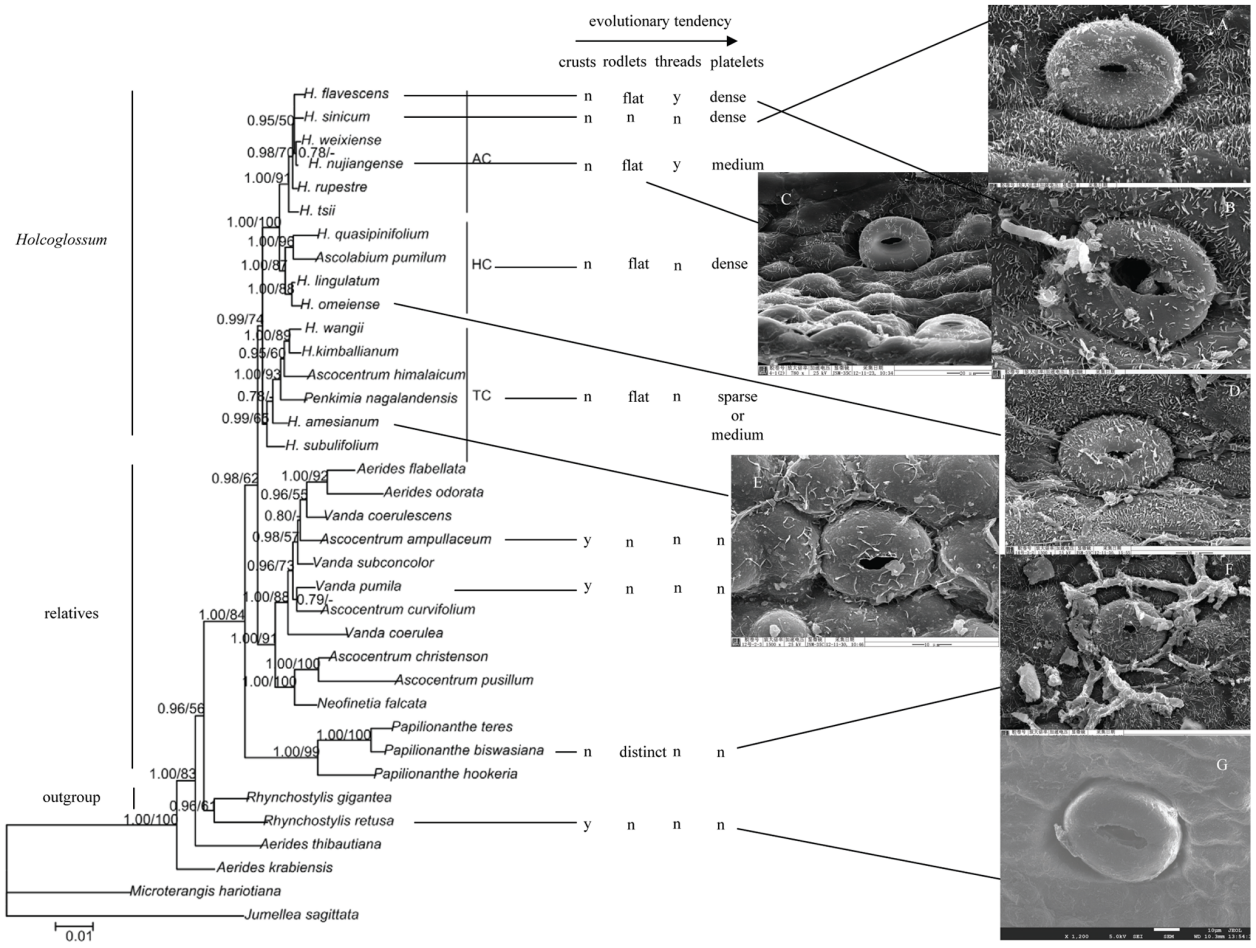
Molecular phylogeny presented by Xiang et al. (2012) with wax types and SEMs of the Ab surface arranged accordingly. A. *Holcoglossum sinicum. B. H. flavescens*. C. *H. nujiangense*. D. *H. omeiense*. E. *H. amesianum*. F. *Papilionanthe biswasiana*. G. *Rhynchostylis retusa*. y represents yes; n represents no.

In the genus *Holcoglossum*, wax types on the Ab surface change and have a general tendency that is consistent with the tendency suggested by molecular phylogenetic studies [13], [20]. The rodlets vary from flat to threads or absent and the platelets vary from sparse or medium to dense in a direction of TC - HC - AC, (Fig. 11). A crust is only found in the outgroup and relatives of *Holcoglossum*; therefore, we suggest that small and dense platelets are more evolutionarily derived than crusts and rodlets (Fig. 11). The wax types vary in a direction of TC – HC – AC, and the latitude increases and the temperature decreases comparatively. Chemical analyses of the wax crystals might provide information that could explain the morphological differences in the wax crystals [35], [36].

### Taxonomic suggestions for *H. himalaicum*

*Holcoglossum himalaicum* is a difficult species with different phylogenetic positions. Recent molecular phylogenetic studies suggest that it is a species of *Holcoglossum* [13] or *Vanda* [37]. In this study, nearly all of the characteristics of the leaf epidermis evaluated under LM are the same as those of the TC clade, including the phylogenetic characteristics for the infrageneric clades of *Holcoglossum* mentioned above (Tables 1 and 2). The wax types of *H. himalaicum* are the same as those of *H. wangii*. Therefore, *H. himalaicum* should be included in the TC clade of *Holcoglossum*.

## Materials and Methods

### Taxon sampling

The mature leaves of 27 herbarium specimens, representing 12 of 17 species of genus *Holcoglossum* [13], were used in this study (Table S1). An additional nine specimens, representing four species from the related genera *Vanda*, *Papilionanthe, Luisia* and *Ascocentrum*, were included for comparison with *Holcoglossum* (Table S1). Another two specimens of *Rhynchostylis retusa* were used as an outgroup, which is according to recent molecular phylogenetic studies [13], [20]–[23], [38] (Table S1). Samples were taken from herbarium specimens.

### Methods

The materials for the LM study were boiled in water before maceration in 35% NaClO. Pieces of leaf epidermis were stained with safranin–alcohol (50%), and then dehydrated in an ethanol series before being mounted in Canada balsam. To verify the constancy of the epidermal structure, at least five slides were assembled from different parts of a single leaf for each sample. Because of the difficulty in controlling the time of maceration in 35% NaClO for all of the species of the AC clades, *Rhynchostylis retusa* and *H. omeiense*, only the wax coats with the prints of the epidermal cells were examined. In addition, certain slides of *H. sinicum* were mounted in water. The leaf samples for the SEM observation were taken from the midsection of the leaf to eliminate variation caused by sampling from different leaf areas. At least three accessions from two or more different collections for each species were sampled to assess the variation within a species. These samples were cut into small pieces ca. 0.5 cm+0.5 cm or ca. 0.5 cm in length, directly attached to stubs without any treatment. After gold sputtering, the samples were examined and photographed under a Hitachi JSM-35C, six samples from the species *Rhynchostylis retusa; H. himalaicum*; and *Luisia magniflora* were examined under a JSM-7001F.

### Terminology

**Figure 12:**
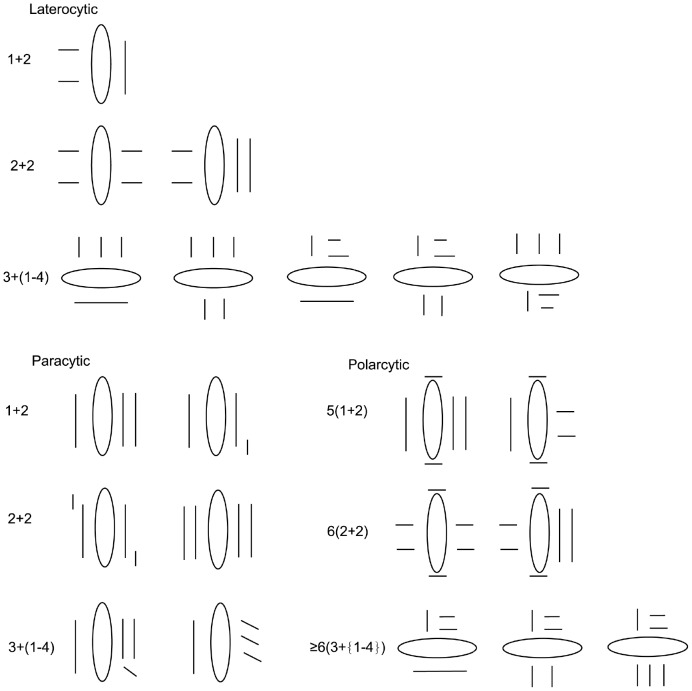
Subtypes of three stomatotypes commonly found in this study. Notes: Every line represents a subsidiary cell and the orientation of its long axis. Its length represents its size, and an ellipse represents a stoma. The notation 1+2 of the laterocytic and paracytic types indicates the distribution of subsidiary cells; in this case, there is 1 on one lateral side of the stoma and 2 on the other side. The notation 5(1+2) of the polarcytic type indicates the distribution of subsidiary cells; in this case, there are five subsidiary cells, with 2 polar and 3 lateral subsidiary cells and 1 on one lateral side and 2 on the other side.

The stomatal terminology is consistent with that of Carpenter [39] because most of the stomatotypes present in this study, such as paracytic, laterocytic and stephanocytic, can be found in Carpenter's work. In addition, several stomatotypes were found with two short polar subsidiary cells and more than two subsidiary cells on at least one lateral side of the stoma, and these types are consistent with the examples from Williams [29], such as 6(4+2) (6 subsidiary cells, with 4 lateral and 2 polar). However, there are similar stomatal type names with different meanings in Carpenter [39], such as laterocytic 1+2 (3 subsidiary cells, with 1 on one lateral side and 2 on the other side). In this study, we named the 6(4+2) type from Williams the polarcytic 6(2+2) type, and it had 6 subsidiary cells, with 2 polar subsidiary cells (not included in the name) and 2 subsidiary cells on each lateral sides. In addition, we increase two subtypes of paracytic type: paracytic 1+2 and paracytic 3+(1–4), and they had the same meaning as the laterocytic subtypes in Carpenter [39]. Illustrations of these types are shown in Figure 12. The terminology for other characteristics was based on the classification of Wilkinson [40], and terminology of the epicuticular waxes was based on Barthlott et al. [35].

## Supporting Information

Table S1 Source of materials.
(DOC)
