# Supplementary material for: Systematic Significance of Leaf Epidermal Features in Holcoglossum (Orchidaceae)
Source: PLoS One. 2014 Jul 1;9(7):e101557. doi: 10.1371/journal.pone.0101557 (PMC4077813; doi:10.1371/journal.pone.0101557)
Supplement: Table S1 — Source of materials. (DOC) [file pone.0101557.s001.doc]

Table S1 Source of material

| Taxon | Locality | Voucher (PE) |
| --- | --- | --- |
| *Holcoglossum amesianum* (Rchb. f. ) Christenson | Yunnan | 00729352 |
|  | Yunnan | X.H. Jin 8086 |
| *H. flavescens* (Schltr. ) Z. H. Tsi JZS | Yunnan | 00729360 |
|  | Yunnan | X.H. Jin 8943-1 |
| *H. kimballianum* (Rchb. f.) Garay | Yunnan | 00729378 |
|  | Yunnan | 00729365 |
|  | Yunnan | 00729376 |
| *H. lingulatum* (Averyanov) Averyanov | Sichuan | 00729385 |
|  | Guangxi | Kadoorie PT 3333 |
|  | Yunnan | 00729378 |
| *H. omeiense* X. H. Jin & S. C. Chen | Sichuan | 00753951 |
|  | Sichuan | 01866870 |
|  | Sichuan | 01866871 |
|  | Sichuan | 00729383 |
| *H. sinicum* Christenson | Yunnan | X. H. Jin 8946 |
|  | Yunnan | 01825258 |
|  | Yunnan | 01825256 |
| *H. subulifolium* (Rchb. f.) Christenson | Hainan | 00729405 |
|  | Hainan | 01866626 |
| *H. wangii* Christenson | Yunnan | 00729406 |
|  | Yunnan | 01855754 |
| *H. weixiense* X. H. Jin & S. C. Chen | Yunnan | Kadoorie PT 3490 |
| *H. nujiangense* X. H. Jin & S. C. Chen | Yunnan | X.H. Jin 6981 |
| *H. rupestre* (Hand. -Mazz.) Garay | Yunnan | X.H. Jin 8998 |
|  | Yunnan | Kadoorie PT 3496 |
| *H. himalaicum* (Deb, Sengupta & Malick) Aver. | Yunnan | Lai Yangjun 11033 |
|  | Yunnan | 01526048 |
| *Ascocentrum ampullaceum* (Roxb.) Schltr. | Yunnan | 00200775 |
|  | Yunnan | 00200778 |
|  | Yunnan | X.H. Jin xishuangbanna |
| *Papilionanthe biswasiana* (Ghose et Mukerjee) Garay | Yunnan | X.H. Jin 8084 |
| *Vanda pumila* Hook. f. | Yunnan | X.H. Jin 6946 |
|  | Yunnan | 01825409 |
|  | Yunnan | Kadoorie PT 682 |
| *Rhynchostylis retusa* (L.) Bl. | Yunnan | 00850333 |
|  | Yunnan | 00729806 |
| *Luisia magniflora* Z. H. Tsi & S. C. Chen | Yunnan | X.H. Jin 6945 |
|  | Yunnan | Lai Yangjun 11028 |
